# Botulinum Toxin in Movement Disorders: An Update

**DOI:** 10.3390/toxins13010042

**Published:** 2021-01-08

**Authors:** Charenya Anandan, Joseph Jankovic

**Affiliations:** Parkinson’s Disease Center and Movement Disorders Clinic, Department of Neurology, Baylor College of Medicine, Houston, TX 77030, USA; canandan@bcm.edu

**Keywords:** botulinum toxin, movement disorders, tremors, dystonia, tics, bruxism, restless legs syndrome, Parkinson’s disease, myoclonus, dyskinesia

## Abstract

Since its initial approval in 1989 by the US Food and Drug Administration for the treatment of blepharospasm and other facial spasms, botulinum toxin (BoNT) has evolved into a therapeutic modality for a variety of neurological and non-neurological disorders. With respect to neurologic movement disorders, BoNT has been reported to be effective for the treatment of dystonia, bruxism, tremors, tics, myoclonus, restless legs syndrome, tardive dyskinesia, and a variety of symptoms associated with Parkinson’s disease. More recently, research with BoNT has expanded beyond its use as a powerful muscle relaxant and a peripherally active drug to its potential central nervous system applications in the treatment of neurodegenerative disorders. Although BoNT is the most potent biologic toxin, when it is administered by knowledgeable and experienced clinicians, it is one of the safest therapeutic agents in clinical use. The primary aim of this article is to provide an update on recent advances in BoNT research with a focus on novel applications in the treatment of movement disorders. This comprehensive review of the literature provides a critical review of evidence-based clinical trials and highlights recent innovative pilot studies.

## 1. Introduction

*Clostridium botulinum*, an anaerobic, rod-shaped bacterium, produces a neurotoxin called botulinum toxin (BoNT) during sporulation [1,2,3]. BoNT is the most potent biological toxin, as it causes botulism manifested by paralysis of muscles and eventual fatal respiratory failure [4,5]. When an action potential arrives at the cholinergic presynaptic nerve terminal, there is an influx of calcium into the presynaptic terminal, which then facilitates acetylcholine vesicle fusion with the presynaptic membrane; this fusion is facilitated by a group of proteins referred to as SNARE (soluble N-ethylmaleimide-sensitive factor attachment receptor) proteins, which include SNAP 25 (25 kD synaptosomal-associated protein) and Syntaxin [6,7]. BoNT acts at the cholinergic presynaptic nerve terminal by cleaving and inactivating SNARE proteins, thus inhibiting release of acetylcholine, which in turn prevents muscle contraction and results in local weakness and paralysis [7,8]. BoNT acts at both the extrafusal and intrafusal muscle fibers, thereby preventing contraction of both agonist and antagonist muscles [8,9]. This biologic effect of BoNT has been turned into an advantage in patients troubled by involuntary muscle contractions, excessive secretions, pain, and other conditions [7]. The paralytic effects of BoNT were initially described in 1817 by Justinus Kerner, a German physician, who suggested that the toxin may be potentially useful in the treatment of St. Vitus’ dance, hypersalivation, and hyperhidrosis [10]. The mechanism of action of BoNT injections to account for the typical 3–4 months of duration has not been fully elucidated, and the original proposal that axonal sprouting occurs at the presynaptic nerve terminal after the injection, after which time the neuromuscular junction integrity is restored when the original nerve terminals regain their exocytic function, hence necessitating repeat injections [11], has been challenged [12].

In 1981, Dr. Jankovic initially injected BoNT into a patient for treatment of blepharospasm (BSP) [10] and subsequently published the results of the first double-blind, placebo-controlled trial of BoNT in cranial–cervical dystonia [13]. The results of this trial, along with additional data, were used by the United States Food and Drug Administration (FDA) to approve BoNT in 1989 for the treatment of BSP and facial nerve disorders such as hemifacial spasm (HFS) [14]. Although only BoNT types A and B have been approved for clinical use by the FDA, there are a total of eight different subtypes: BoNT A to H [6].

There are currently four FDA approved BoNT formulations: the three types of botulinum toxin type A (BoNTA) available are onabotulinumtoxinA (Botox; Allergan, CA, USA), abobotulinumtoxinA (Dysport; Ipsen-Pharma, UK), and incobotulinumtoxinA (Xeomin; Merz Pharma, Germany); rimabotulinumtoxinB (Myobloc in the USA; Supernus Pharmaceuticals, Inc, Rockville, MD; Neurobloc in Europe, Sloan Pharma, Switzerland) is a BoNTB preparation [6,10].

There are several BoNT preparations that are currently in development but have not yet been approved by the FDA. DaxibotulinumtoxinA is a novel BoNTA preparation that was recently evaluated in a phase 3 trial (ASPEN-1) in cervical dystonia (https://www.businesswire.com/news/home/20201014005360/en/). This study enrolled 301 patients from 60 sites in the U.S., Canada, and Europe and confirmed the findings of an earlier phase 2 study [15] in that it found that daxibotulinumtoxinA is safe and effective. Interestingly, at doses of 125 U, it has a median duration of effect (based on the median time to loss of 80% of the peak treatment effect) of 24 weeks. This relatively long duration of action offers potential advantage over other formulations in that it may allow increasing the intervisit interval beyond the conventional 3–4 months. LanbotulinumtoxinA (Prosigne; Shanghai, China) is a new preparation of BoNTA marketed chiefly in Asia [16,17].

The doses of different formulations are not interchangeable, but based on prior studies, the following ratios are often used in clinical practice when switching from one to another BoNT product to achieve similar results: onabotulinumtoxinA:incobotulinumtoxinA = 1:1; onabotulinumtoxinA:abobotulinumtoxinA = 1:2.5, and onabotulinumtoxinA:rimabotulinumtoxinB = 1:50 [10].

With long term use of BoNT, there is a risk of developing neutralizing antibodies (NAbs) [18], and patients may stop responding to BoNT. Factors that increase the risk of developing resistance to BoNT include a high protein load in some formulations, large individual and cumulative doses of BoNT, and short intervisit intervals, especially booster injections [18,19,20,21,22,23]. Immunogenicity varies among the different products and has been reported as low as 0% for incobotulinumtoxinA and as high as 42.4% for rimabotulinumtoxinB [20]. Brin et al. [23] noted a 1.2% frequency of NAbs based on mouse protection assay (MPA) in patients treated for cervical dystonia with onabotulinumtoxinA. In contrast, Albrecht et al. [18] reported a prevalence of 14% of NAbs in 596 treated with BoNTA, mostly abobotulinumtoxinA, for a mean of 5.3 years based on mouse hemidiaphragm assay (MHDA). Since biological assays such as MPA and MHDA are difficult to perform and they involve sacrificing animals, there is a huge unmet need to develop a simple, inexpensive, sensitive, and specific test for BoNT-blocking antibodies. If a patient reports lack of improvement (less than 25%) after at least two or three consecutive treatment visits, this raises a high level of suspicion of immunoresistance [20]. In this case, unilateral brow injection may be performed as a clinically useful test [20]. This involves injection of BoNT in the right medial eyebrow and reassessing in 1–2 weeks for paralysis of the right procerus/corrugator as manifested by asymmetric frowning, which would disprove immunoresistance [20]. We have not included spasticity in this article, as we believe it is beyond the scope of this review. The reader is referred to some recent reviews on this topic [24,25,26].

**Figure 1:**
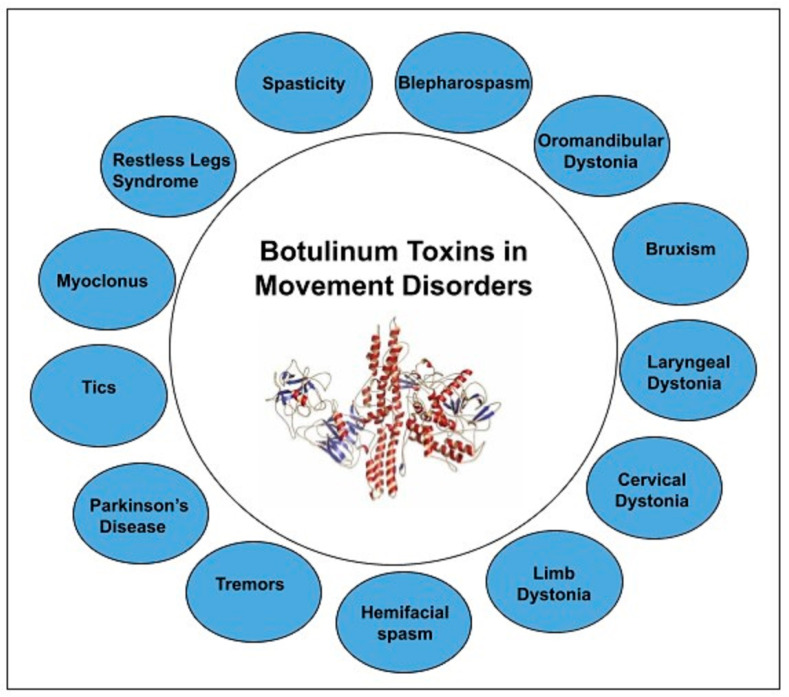
BoNT in Movement disorders.

Our manuscript provides a comprehensive review of botulinum toxin in movement disorders. Figure 1 depicts the variety of movement disorders where botulinum toxin is used for therapeutic purposes.

## 2. Discussion of BoNT Use in Different Indications

### 2.1. Dystonia

Dystonia is defined as a movement disorder characterized by sustained or intermittent muscle contractions causing abnormal, often repetitive, patterned movements, postures, or both [27,28].

It is frequently associated with activity but may also be present at rest and worsens with stress, anxiety, and fatigue [29]. The prevalence has been reported to range between 15 and 225 per 100,000 individuals [30].

We conducted a PubMed search on 28 May 2020; using the title words botulinum and dystonia, a total of 438 articles were identified. 340 of these were in English and were human studies. Of the 340 articles, 71 were review articles, 154 were either prospective or retrospective trials, 32 were randomized controlled trials (RCTs), 58 were case reports, 6 were commentaries, 8 were unavailable for review, and 11 articles were irrelevant. The clinical composition of 340 articles were different types of dystonia: 2 axial, 1 blepharospasm (BSP), 49 multiple types, 186 cervical dystonia (CD), 1 cranial, 15 unspecified, 17 laryngeal, 28 limb, 5 lingual, 30 oromandibular dystonia (OMD), 1 torsion, and 5 tardive.

#### 2.1.1. Blepharospasm

Blepharospasm (BSP) is a form of focal dystonia [31,32] that is characterized by involuntary and prolonged bilateral contraction of periorbital muscles, including orbicularis oculi (OOc), procerus, and corrugator [33]. Its estimated prevalence is 5 per 100,000 people [16,30,34,35]. Though it may start with increased blinking, it can progress to disabling closure of the eye due to involuntary contractions of OOc [36]. The resulting blindness makes activities of daily living such and reading, walking, and writing hard to perform [33].

We conducted a PubMed search on 21 April 2020 using the botulinum and BSP as title words; a total of 213 articles were identified. 155 of these were in English and were human studies. Of the 155 articles, 17 were review articles, 68 were either prospective or retrospective trials, 14 were RCTs, 11 were case reports, 6 were commentaries, 10 were unavailable for review, and 29 articles were irrelevant.

Early studies including Frueh et al. [37] and Tsoy et al. [33] described 16 and 43 patients with BSP, respectively, who were injected with BoNTA and noted marked relief of eyelid spasms. There are several other open-label studies [38,39,40] that documented BoNTA to be an effective treatment option, with an average duration of effect of 2.5–2.8 months. The side effects included transient tearing (5–10%), ptosis (5.4–9%), dry eyes (3–7.5%), blurred vision and/or diplopia (1–2%), ectropion (1%), and photophobia (2%) [36,41,42,43]. In our early series of 42 BSP and 115 CD patients who received BoNTA and were followed prospectively for a minimum of five follow-up visits, the CD patients were slightly younger, needed higher dose of BoNT (218 vs. 44 units), had longer latency (6.9 vs. 3.9 days), but similar total duration of effect (15.5 vs. 15.8 weeks) and frequency of complications (26.6% vs. 32.8%) [44]. Another study involving 178 BSP patients treated with BoNTA, initial dose 2.5 in each of four sites per eye, with long-term follow-up showed 93% symptom relief, 3.6 months mean duration of effect, 1.7% remission of symptoms, and no systemic side effects [45]. Pretarsal injections are generally recommended because of more robust benefit and less frequent ptosis compared to preseptal injections [46,47].

The American Academy of Neurology (AAN) published updated practice guidelines in 2016 regarding the use of BoNT in multiple movement and neurologic disorders [48] and found that: (i) onabotulinumtoxinA and incobotulinumtoxinA were probably effective for BSP treatment (Level B), and (ii) abobotulinumtoxinA was possibly effective (Level C). The Cochrane database review in 2005 stated that though randomized controlled data were unavailable, based on case control and cohort studies, BoNT was determined to be beneficial in 90% of patients with BSP [49]. A more recent Cochrane database review included three RCTs and concluded with moderate certainty that a single injection of BoNTA reduced blepharospasm severity [50].

The following are the randomized trials of BoNT in the treatment of BSP and BoNT (Table 1). Lolekha et al. [34], Wickwar et al. [51], Wu et al. [52], Quagliato et al. [53], Price et al. [54], and Li et al. [55] will be discussed under hemifacial spasm (HFS). Although the vast majority of patients are satisfied with the effects of BoNT, many patients note that their symptoms recur before their next injection. The need for BoNT preparations with longer lasting effects will be further highlighted in the section on CD.

#### 2.1.2. Oromandibular Dystonia

Oromandibular dystonia (OMD) is a focal dystonia that affects face, mouth, and jaws [29]. This can be jaw opening, closing (also discussed with bruxism), or lingual (discussed separately) dystonia [66,67,68]. Prevalence is 30–70 per 100,000 patients and has a female predominance [29,66,68]. Age at onset is in the 5th or 6th decade. Jaw opening OMD is likely due to contraction of lateral pterygoid, mylohyoid, thyrohyoid, infrahyoid, and geniohyoid with posterior digastric [14,69].

Tan and Jankovic [70] studied 162 OMD patients who had been followed for about 10 years (mean age 57.9 ± 15.2 years). The patients were injected with onabotulinumtoxinA in the submentalis muscle complex (mean dose 28.6 ± 16.7 units per side) in patients with jaw opening OMD and in masseters (54.2 ± 15.2 U per side) in patients with jaw clenching with or without bruxism. The duration of benefit was 16.4 ± 7.1 weeks and 31.5% experienced adverse effects (dysphagia 10.2%, dysarthria 0.9%) [70]. A retrospective chart review from 1995–2011 included 59 OMD patients, showed inter-BoNT injection interval of 3.8 months; 47.5% had jaw closing, 35.6% opening dystonia, and 16.9% were jaw lateral deviation OMD [71]. A survey involving 14 patients who had received BoNT for OMD for 8–10 years revealed that 9 of 14 patients had continued with BoNT treatment with and without other oral agents [29]. An intraoral approach for BoNT injection in six of the eight patients with OMD showed significant improvement; only one had adverse effect of nasal speech [72].

In a systematic review [73] involving 387 OMD patients treated with BoNT, 27.1% had side effects, most frequently dysphagia. Comella et al. has performed systematic review of BoNT in OMD [66] and concluded that “that BoNT may be the most effective treatment available, with improvement in movement and quality of life in OMD”.

Novel injection techniques with use of ultrasound and CT images fused with plaster cast and software assisting accurate needle insertion in lateral pterygoid improved outcomes in jaw opening dystonia (66.3% and 54.4% with and without computer aided design guidance, respectively) [74].

#### 2.1.3. Bruxism

Bruxism is a condition characterized by clenching of the jaw and biting/grinding of the teeth that occurs mainly during sleep [14]. Some of its symptoms are similar to oromandibular dystonia [75]. This can lead to damage to teeth, headaches, and temporomandibular joint problems [14]. It can be seen in some diseases such as sleep apnea, PD, Huntington’s disease (HD), and some autism spectrum disorders (ASD), and muscles most involved in this seem to be masseter, temporalis, and medial pterygoid [14,76]. “Bruxism” and “botulinum” were used as title words for the PubMed search. 27 articles were present; of these, 27 were in English and human studies. Of the 27 articles, 6 were review, 7 were either prospective or retrospective trials, 4 were RCTs, 9 were case reports, and 2 were commentaries.

Earliest case reports of BoNT for bruxism management was in 1990 and 1997 [77,78]. In a study of 18 patients who had bruxism for about 14.8 ± 10 years, the patients were injected with BoNTA in the masseters with a mean dose of 61.7 ± 11.1 MU to each side [79]; this resulted in near abolishment mean score of 3.4 ± 0.9 (total abolishment = 4) with only one patient reporting dysphagia. A recent systematic review that selected studies that assessed efficacy of BoNTA in the treatment of bruxism based on bite force or EMG of masseter concluded there was not enough evidence to treat bruxism with BoNTA [80]. However, another systematic review of BoNTA in bruxism reviewed six RCTs and four case series concluded that BoNTA was effective for the treatment of bruxism [81]. In our experience, we start at about 25 U of onabotulinumtoxinA in each masseter, but may increase the dosage up to 300 U to include the masseters and temporalis muscles for optimal benefit and to control or prevent further bruxism [14]. Table 2 lists RCTs associated with BoNT and bruxism.

#### 2.1.4. Lingual Dystonia

Lingual dystonia is a rare type of focal dystonia with estimated prevalence of about 4% of dystonic population [86]. It is usually a debilitating type of focal dystonia frequently brought on by talking and eating noted in patients with neurodegenerative disorders such as neuroacanthocytosis or neurodegeneration with brain iron accumulation [87]. It may also be a manifestation of tardive dyskinesia [86,88,89]. We conducted a PubMed search on 28 May 2020; using the title words botulinum and dystonia, a total of 438 articles were identified. 340 of these were in English and were human studies. Of the 340 articles, five were lingual dystonia. Of these, one was unavailable for review, two were retrospective studies, one was prospective, and one was a review article.

A retrospective study of 172 patients divided lingual dystonia into four types: protrusion (68.6%), curling (7.6%), retraction (16.9%), and laterotrusion (7%) [87]. BoNT was administered to 136 patients, most of whom noted some improvement in mastication, pain, and phonation [87]. Mean dose was 43.1 ± 5.3 units. Transient trouble with swallowing occurred in 12.5% of patients. In another study, 50 units of abobotulinumtoxinA injected into each genioglossus was effective in treating lingual-dystonia-related tardive dyskinesia [89]. In a series of 30 patients with lingual dystonia who participated in a QoL survey, OMD questionnaire-25 (OMDQ-25) score dropped from 46.8 ± 17.8 pre-BoNT to 38.2 ± 17.6 post-BoNT (*p* = 0.004) [86]. Dysphagia occurred in 16.7% of patients. A retrospective chart review revealed 4% of lingual dystonia (17 patients; 5 cranial dystonia, 2 primary generalized dystonia, 7 tardive dystonia, 1 each of heterodegenerative, multiple causes, postinfectious) in 421 dystonia patients, and 9 of 17 had BoNT injections; 55.6% had improvement and 1 patient developed dysphagia requiring gastrostomy tube placement [88].

#### 2.1.5. Laryngeal Dystonia

Spasmodic dysphonia is a type of laryngeal dystonia manifested by action-induced sustained contraction of the vocalis muscles leading to approximation or separation of the vocal folds resulting in abnormal phonation [90,91]. Spasmodic dysphonia can be due to adductor spasm (this is more common and manifests as strained voice) or, less commonly, abductor spasm (from posterior cricoarytenoid muscle spasm leading to breathy or whispering voice) [90,92]. Studies showed that 16–32% of patients had primary laryngeal dystonia that can spread to other body parts [91,93] and 12.1% [94] had family history of dystonia.

We conducted a PubMed search on 28 May 2020; using the title words botulinum and dystonia, a total of 438 articles were identified. Of those, 340 of these were in English and were human studies. Of the 340 articles, 17 were laryngeal dystonia. Of these, 1 was a review article, 6 were case reports, and 10 were either prospective or retrospective studies.

A chart review of 155 patients with adductor spasmodic dysphonia who received thyroarytenoid injections found that the mean duration of beneficial effect from local BoNT injection was 13–14 weeks, and this was not influenced by age or gender of the patient [95]. A retrospective review of 900 laryngeal dystonia patients who had undergone EMG-guided BoNTA injections for the treatment of adductor or abductor spasmodic dysphonia showed that 90% and 66.7% benefited for an average of 15.5 weeks [91]. A Cochrane review concluded that the randomized controlled trials were not adequate enough to arrive at an unbiased conclusion about the effects of BoNT [96].

#### 2.1.6. Cervical Dystonia

Cervical dystonia (CD) is the commonest form of focal dystonia in a movement disorder clinic [6]. It is manifested by involuntary contractions of multiple cervical muscles, leading to abnormal postures and movement in the neck, often associated with tremor and pain. Its prevalence is estimated to range around 5.7–400 per 100,000 persons [97,98] and usual age at onset is 40 [99]. A recent systematic review quoted that 15.4% of patients with CD, particularly the younger ones, report remission, typically occurring 4.5 years after onset of dystonic symptoms, but the majority (63.8%) eventually relapse [100].

Patients with CD often exhibit dystonic head tremor and a variety of different postures [101]. Anterocollis is the most difficult CD posture to treat with BoNT; as a result of this, patients with this form of CD are often excluded from clinical trials. We, however, found that some such patients benefit from injections of the sternocleidomastoid (SCM), anterior scalenus, submental complex, longus coli, and longus capitis, although the latter requires ultrasound or other imaging techniques in addition to EMG guidance [69,102]. Initial doses of around 10–25 U of onabotulinumtoxinA and injection in the lower portion of SCM helps avoid side effects such as dysphagia [14]. The Toronto Western Spasmodic Torticollis Rating Scale (TWSTRS) scale is used most frequently as an outcome measure in most clinical trials of CD [103].

In one of the largest studies, 616 patients with CD underwent BoNTA (abobotulinumtoxinA) injections with mean dose of 246–255 U in SCM, 423–445 U into splenius capitis, 227–242 U into trapezius, and 152–156 U into levator scapule; latency to benefit was about 7.5 days, maximum effect was between 7–35 days postinjection, and total effect lasted 11 ± 2.4 weeks [104]. In another study, 207 patients with CD received BoNTA for 6.7 ± 3.5 years; the mean dose of abobotulinumtoxinA was 389 ± 144 U and the mean dose of onabotulinumtoxinA was 145 ± 44 U [105]. Mean doses of abobotulinumtoxinA/onabotulinumtoxinA in splenius capitis, levator scapulae, trapezius, and SCM was 160/50, 70/25, 100/40, and 60/25 U, respectively. Latency to effect was around 7.6 ± 3.5 days (abobotulinumtoxinA), and side effects such as neck weakness, dysphagia, and pain occurred at 5%, 8%, and 9%, respectively. Latency to effect was 7.7 ± 3.3 (onabotulinumtoxinA) and adverse effects such as neck weakness, dysphagia, and pain occurred at 7%, 9%, and 6%, respectively. Systemic side effects were not noted in either formulation. Less than 2% developed NAbs [105]. In 326 patients with CD who prospectively received BoNTA (onabotulinumtoxinA) with a median of nine sessions, 1.2% (four patients) tested positive for NAbs using mouse protection assay (three of four did not respond to BoNT and one still experienced benefit) [23].

A retrospective analysis of 89 patients (51 CD, 34 BSP, and 26 OMD) showed that BoNT remained effective and safe for the treatment of these conditions over 20 years [106]. Castelao et al. conducted a systematic review, which included eight RCTs and concluded that BoNTA was safe and effective in the treatment of CD. Doses ranged from 150 U to 236 U of onabotulinumtoxinA, 120 U–240 U of incobotulinumtoxinA, and 250–1000 U of abobotulinumtoxinA. BoNTA led to a mean 8.1 point reduction in TWSTRS total score at four weeks post-BoNTA [107]. Cochrane reviews showed that: BoNTA and BoNTB were effective for treatment of CD [108,109], and that BoNTA was more effective than trihexyphenidyl [110].

Dysphagia, which may be present in up to 25% of patients with CD even before initial BoNT treatment [111], is one of the most frequent side effects, occurring in 9% of treated patients, followed by neck weakness, which occurred in 10% [107]. In a prospective study of 18 patients with CD, in additional rotation, tilt, forward shift, backward shift, shoulder elevation, shoulder depression, and tremor, 9 (50%) complained of coughing and/or choking and this increased to 11 (61%) along with voice changes in 9 (50%), and sensation of food stuck in the throat in 8 (44%) [111].

Though BoNT in CD is efficacious, it sometimes is not effective and has adverse effects of weakness, this can be mitigated to some extent using ultrasound guidance [112]. Of 98 EMG guided injections for CD, 34.7% were associated with dysphagia; however, when injected with ultrasound and EMG guidance, the risk of dysphagia was apparently reduced to 0% after 27 injections [97]. Polymyographic EMG can help increase effectiveness of BoNT in CD; however, more studies are necessary to determine whether EMG-guided injections clearly provide incremental benefit to justify increased pain, time, and cost compared to palpation and surface anatomy [98]. Certainly, ultrasound and EMG-guided injections are necessary when approaching deep neck muscles.

A Cochrane review in 2016 included four RCTs in CD and found a 14.7% improvement with BoNTB [113]. A Cochrane review that included four RCTs concluded that there was low-level evidence that first injection with BoNTA (onabotulinumtoxinA) as compared to BoNTB (rimabotulinumtoxinB) was similar in treatment of CD [114]. A study with 40 CD patients showed that onabotulinumtoxinA and incobotulinumtoxinA had similar efficacy at 1:1 conversion [115]. A multicenter study with 100 CD patients showed that about 1/3 of patients who were BoNTB NAbs negative pre-BoNTB became positive, suggesting increased antigenicity with BoNTB [20,116].

Several studies have evaluated the changes in the neuronal network in CD patients post-BoNT [117,118]. A study involving 17 patients with CD used fMRI pre- and 6 months post-BoNT [118]. CD patients showed higher activity in the basal ganglia and thalamus at baseline but after three sessions of BoNT in 6 months, and the connectivity between thalamus and basal ganglia was lower [118]. Another study evaluated 12 CD patients pre- and 4 weeks post-BoNT and found that BoNT injection alters sensorimotor activation [119]. Mahajan et al. [120] found changes in multiple brain areas, including the left putamen and right superior parietal gyrus on magnetoencephalography post-BoNT in CD. Pre- and post-BoNT studies showed an increase in activity in certain cortical areas involved in motor and cognitive planning, indicating that alterations in peripheral input can lead to changes in central nervous system processes [121]. In seven patients with CD who had functional MRI, pre-BoNT in CD when the median nerve was stimulated showed activation of contralateral primary but not the secondary somatosensory cortex; four weeks after, BoNT normalized and was similar to the nine controls showing activation of the primary and secondary cortices [122]. This study concluded that there is widespread somatosensory physiology disruption.

Common causes of BoNT discontinuation in CD is lack of response, short duration of benefit, side effects, inconvenience, and cost [123,124]. In one survey, of 209 respondents, the mean reported onset of BoNT-A therapeutic effect was 11.7 days and the time to peak effect was 4.5 weeks; the time from injection to symptom re-emergence was 73.6 days (~10.5 weeks); 88% experienced symptom re-emergence between injections [125]. Therefore, developing new BoNT preparations with longer duration of action is one of the highest priorities in experimental therapeutics of dystonia. In this regard, refer to the above discussion about daxibotulinumtoxinA.

Of 216 patients with CD who were determined to be secondary nonresponders (SNR), based on retrospective chart review compared to three patients with CD who had responded to BoNT, it was determined that prior surgery, prior side effects of BoNT, physical therapy, prior antipsychotic use, and high mean dose of BoNTA were linked to SNR [126]. Based on chart review of CD patients, it was observed that those who had their first BoNT injection between 1989 to 2000 had a gradual increase in required dose and 19 became nonresponders [127]. In a retrospective review of 118 patients, in long-term follow-up, the median Tsui score was higher in responders and they also had a higher rate of taking oral medications [128]. The rates of employment improved after BoNT for CD [129]. Based on retrospective chart review of 568 patients treated with abobotulinumtoxinA for about 13 years, partial secondary treatment failure occurred in 1.6% per year, or about 14.5% in 9 years [130]. AAN published updated practice guideline in 2016 regarding the use of BoNT in multiple movement and neurologic disorders [48]: (i) abobotulinumtoxinA and rimabotulinumtoxinB were effective for CD treatment (Level A), and (ii) onabotulinumtoxinA and incobotulinumtoxinA were probably effective (Level B). Table 3 discusses the various randomized trials involving BoNT and CD.

#### 2.1.7. Limb Dystonia

Upper limb dystonia typically presents as dystonic writer’s cramp, task-specific musician’s dystonia, or other occupational or sports dystonia [155,156,157,158]. Lower limb dystonia can present as foot dystonia (as seen in PD) or performance dystonia (e.g., runner’s dystonia) [159]. Sometimes one can observe overflow into adjacent muscles or mirror movements on the opposite side (using the unaffected hand to write can produce dystonic positions in the affected hand) and careful evaluation of this phenomenon can be helpful in selecting the appropriate muscles to target with BoNT [160]. Writer’s cramp is probably the most common form of dystonia, affecting about 21 people per 100,000 [161]. In a double-blind RCT of BoNTA involving 40 patients with writer’s cramp, 70% and 31.6% in the BoNT and placebo groups reported improvement, respectively (*p* = 0.03); hand weakness was reported by 18 (90%) patients in the BoNT-A group despite EMG recording in selected muscles [162]. Another study showed that 12 of 20 patients had improved pen control after BoNT [163]. In a series of 84 patients with musicians with dystonia, 69% noted improvement after BoNT, but 98% noted some weakness [164]. BoNT is found to be effective in dystonia associated with corticobasal syndrome (CBS) [165].

We conducted a PubMed search on 28 May 2020; using the title words botulinum and dystonia, a total of 438 articles were identified. Of those, 340 of these were in English and were human studies. Of the 340 articles, 28 limb dystonia. Of these, 16 were prospective/retrospective studies, 7 were case reports, 2 were review articles, and 3 were RCTs.

In a retrospective series with 20 patients with focal hand dystonia treated for 10 years with BoNT, it was concluded that that BoNT was a safe and fruitful treatment [166]. Nine patients had writer’s dystonia, five had musician’s dystonia, one had typing dystonia, and five had mixed dystonia. EMG-guided injections were performed; mean dose required was higher later in the years (49.9 units) as compared to 24.9 units early on [166], and antibody resistance was not noted. One study showed that haptic (touch) manipulation technique can significantly enhance the effectiveness of BoNT-A therapy and improve dystonic writer’s cramp [167]. Cole et al. [168] also performed a double-blind RCT of BoNTA in 10 patients with focal hand dystonia and noted subjective improvement in 8 of the 10 patients treated with BoNT. In another study involving 12 patients with writer’s cramp randomized to receive only EMG-guided BoNT therapy or BoNT and occupational therapy, the patient-rated subjective scale scores at 20 weeks were not significantly different between the two groups [158]. There was a significant decrease (28%) in writer’s cramp impairment scale, but the primary endpoint, patient-rated subjective scale, was not achieved. In a survey involving self-administered questionnaires in 42 patients, 10 patients with BSP, 19 with CD, and 13 with focal hand dystonia the mean benefit for the entire population was 66.0% and weakness occurred in 20.5%; patients with hand dystonia had the longest maximum benefit (mean duration 87.5 days) [169].

In a series of 14 patients with foot dystonia, pre- and post-BoNT, gait assessment showed improvement in stride and step length, balance, and gait velocity [159]. In six patients with foot dystonia associated with PD, about 250–400 units were injected in the affected muscles resulting in meaningful relief of pain, dystonia, and gait [170]. There are several case reports and series [171] of foot dystonia in PD that improved after BoNT. In one study, onabotulinumtoxinA was injected into tibialis posterior or anterior, gastrocnemius, flexor digitorum longus, and extensor hallucis longus in 27 patients with marked improvement in spasms and pain [171]. Table 4 discusses the various randomized trials involving BoNT in limb dystonia.

### 2.2. Hemifacial Spasm

Hemifacial spasm (HFS) is a peripherally induced unilateral facial movement disorder characterized by irregular, clonic, or tonic contractions of muscles innervated by the ipsilateral facial nerve. Its estimated prevalence is around 10 in 100,000 [174,175]. The condition usually begins as spasms of lower eyelid on one side of the face, which eventually spreads to upper eyelid and other muscles in ipsilateral face, often associated with elevation of ipsilateral eyebrow referred to as the “other Babinski sign” [34,176]. The estimated prevalence is 14.5 and 7.4 per 100,000 in women and men, respectively [174]. Primary HFS is thought to be related to compression of the facial nerve at the exit zone by an aberrant blood vessel loop. Secondary HFS is related to prior facial nerve injury or Bell’s palsy or brain stem damage [177]; 76% and 21% of HFS are primary and secondary respectively [178]. There is some evidence that facial motor nucleus excitability is reduced after BoNT injections [179].

We conducted a PubMed search on 21 April 2020. Using botulinum and hemifacial as title words, we identified 157 articles; of these, 118 were in English and were human studies. Of the 118 articles, 9 were review articles, 74 were either prospective or retrospective trials, 13 were RCTs, 5 were case reports, 6 were commentaries, and 11 articles were irrelevant.

In 1985, Savino et al. published one of the earliest case series in 15 patients who experienced relief of HFS after BoNT injections [180]. In a series of patients with BSP (*n* = 70), HFS (*n* = 13), CD (*n* = 195), hand dystonia (*n* = 22), and oromandibular dystonia (*n* = 45) who underwent BoNT injections, 94%, 92%, 90%, 77%, and 73% experienced relief of their symptoms, respectively [181]. In another series, 98% of 130 patients with HFS patients experienced relief of symptoms after BoNT injection [182]. In a retrospective review of 100 HFS patients who were treated with a mean dose of 28 U of onabotulinumtoxinA and were followed for 4 years, showed a mean duration of effect of around 3.1 months and latency to onset of effect of 7.1 days [183]. There are numerous prospective and retrospective trial which evaluated the use of BoNTA injections that showed safety and benefit in patients with HFS [184,185,186,187,188,189]. Cakmur et al. evaluated pretarsal versus preseptal injections in 28 and 25 patients with HFS and BSP, respectively, and found that pretarsal BoNTA had better relief of symptoms, longer duration of effect, and lower incidence of ptosis [190]. Results from another study of 72 HFS and 38 BSP patients with a crossover design concluded that pretarsal and preseptal injections provided similar beneficial effects; however, the pretarsal group had longer duration of benefit [191]. A systematic review that was published recently stated that they did not identify RCTs of BoNTA in HFS [192]. In our practice, we inject mainly in the pretarsal portion of the orbicularis oculi in patients with BSP and HFS. Cochrane review (based on a single study with study size = 11) concluded that the benefit rate of BoNT in HFS was between 76–100% and that due to this effect size, it would be extremely hard and unethical to conduct new placebo-controlled trials with a large sample size [193].

Side effects of BoNT for HFS include ptosis (7.8–36%), double vision (1.6%), blurred vision (2.5%), dry eyes/exposure keratitis (2.5%), dysphagia (5.5%), facial droop (3.5–5.5%), eye lid swelling/ecchymosis (3.8%), nausea (2.5%), and conjunctival redness [190,194,195].

Tunc et al. [196] assessed BoNT injections efficacy in 69 patients with idiopathic HFS (*n* = 46) and those with HFS due to definite neurovascular compression (*n* = 23) and found that those with idiopathic HFS had more robust improvement with BoNT. Although some favor surgical vascular decompression as a treatment of HFS, most neurologists prefer BoNT, as there is a lower risk of permanent adverse effects such as facial paralysis and deafness [197]. Table 5 discusses the various randomized trials involving BoNT and HFS.

### 2.3. Tremors

Tremor, an involuntary, rhythmic, oscillatory movement of a body part, is the most common movement disorder in a movement disorder clinic [204,205]. When oral medications do not adequately control the tremors, as is the case in 30% of patients with essential tremor (ET), BoNT should be considered as a therapeutic option [206]. We conducted a PubMed search on 9 July 2020; using botulinum and tremor as title words, a total of 49 articles were identified. Of those, 43 of these were in English and were human studies. Of the 43 articles, 4 were review articles, 18 were either prospective or retrospective trials, 8 were RCTs, 12 were case reports, and 1 was commentary. The clinical subsets of the 43 articles were 8 ET, 2 ET PD, 3 jaw tremor, 6 palatal, 5 multiple tremor types, 9 vocal, 1 each for PD, tremor/tic, orthostatic, head tremor with CD, neuropathy-associated, head tremor, and multiple sclerosis associated with Holmes tremor.

In 1981, Jankovic and colleagues reported the earliest series of 51 patients with different tremor types who benefited from BoNT [207]. Trosh and Pullman published a prospective study with 26 patients (12 and 14 of PD and ET, respectively) who also benefited from BoNT [208]. Fixed doses, limited muscles being injected, and complicating weakness postinjections initially made BoNT use for tremors unsatisfactory [205]. In 2015, a series of 28 PD patients with tremors underwent muscle selection of incobotulinumtoxinA and patients improved at 16 weeks [209]. In an open-label prospective trial, 31 ET patients received 3 cycles of BoNTA based on kinematic analysis guided muscle selection and dose administered [206]; it showed that BoNTA reduced tremor by 47.7% at 6 weeks and the improvement lasted 18–30 weeks. In a series of 10 patients with ET who received BoNTA using kinematics every 16 weeks, a 33.8% functional improvement was noted when selected muscles were injected [210]. The series was later expanded to include 28 PD and 24 ET patients who were injected with BoNTA using computer-based kinematics [210].

Mittal and Jankovic (2019) had provided a systematic review of BoNT in tremors [204] and concluded that most studies were open-label and that there was a need for well-designed controlled trials of BoNT in the treatment of ET and PD tremors. In a retrospective analysis by Niemann and Jankovic [211] of 91 patients (53 ET, 31 dystonic, 9 PD, 1 cerebellar), 81.3% of whom received injections into flexor carpi radialis or ulnaris (mean dose per limb 71.8 units of onabotulinumtoxinA), only 12.2% had transient weakness. This is in contrast to earlier double-blind, placebo-controlled studies by Jankovic et al. [212] and Brin et al. [213], during which the wrist extensors were also injected and, as a result, many patients experienced finger extensor weakness. Therefore, we no longer inject the extensor hand muscles [211]. In a series of 19 patients with proximal tremors, injections in muscles such as supra/infraspinatus, teres major/minor, biceps, triceps, deltoid, and pectoralis major resulted in at least moderate benefit in 63%, but 15% had no benefit [214]. In 20 patients with severe ET, BoNT (mean total dose 95.5 ± 40.58 per patient) improvement was noted in activities of daily living and in severity tremor scale [215]. The investigators also concluded that excluding extensor carpi muscle did not affect efficacy of BoNT. Table 6 lists RCTs associated with tremors and BoNT.

### 2.4. Parkinson’s Disease

PD is a neurodegenerative disease with incidence around 118 per 100,000 person years [223]. There are a variety of symptoms in PD that have been amenable to the treatment with BoNT including hand tremors, jaw tremors, axial dystonia, rectal dystonia, freezing of gait, sialorrhea, and levodopa-induced dyskinesias [14,204,224]. We conducted a PubMed search on 11 July 2020; using botulinum and Parkinson as title words, a total of 58 articles were identified. Of these, 49 of these were in English and were human studies. Of the 49 articles, 7 were review articles, 19 were either prospective or retrospective trials, 14 were RCTs, 7 were case reports, 1 was unavailable for review, and 1 was a commentary. Table 7 lists PD-related conditions amenable to BoNT treatment. Table 8 lists RCTs associated with PD and BoNT.

#### 2.4.1. Camptocormia

Flexion of the trunk (camptocormia) may be caused by a variety of etiologies, including axial dystonia, abnormal posture related to PD or other Parkinsonian disorders, and extensor myopathy [236]. Dystonic camptocormia may improve with BoNT injections. Using title words botulinum and camptocormia in a PubMed search, five articles that were human trials were identified in English. Of these, one was review, one was prospective/retrospective, one was case series, and two were commentaries.

In our experience and in the experience of others [237,238], injections of BoNT into rectus abdominus or the external abdominal oblique muscles seems most helpful [14]. A case series of four patients with PD and camptocormia who received ultrasound-guided botulinum injections of 500–1500 MU in iliopsoas failed to show improvement in camptocormia [237]. In another series involving 10 patients with Parkinsonian camptocormia who received incobotulinumtoxinA 100–300 U in either rectus abdominis or iliopsoas, both groups failed to show substantial improvement [239]. In a review of BoNT in camptocormia related to PD and other movement disorders, Bertram et al. [240] concluded that the evidence for the use of BoNT in paravertebral and truncal muscles is not sufficient to make any definitive recommendations. In five patients with extensor truncal dystonia (opisthotonus, which is opposite of camptocormia), BoNT using doses of 25–50U injected into each of the 4 to 6 lumbar paravertebral muscles provided meaningful improvement in three patients [241].

#### 2.4.2. Sialorrhea

Botox (20–50 MU) was injected into each parotid in 18 patients with PD and drooling, and all patients had improvement of symptoms in 4–6 days [231]. Possible complications include dry mouth, dysphagia, infection, and hematoma. In another open series with 16 PD patients with drooling, the patients experienced relief of symptoms post-BoNT [242]. Use of ultrasound for salivary gland injections is thought to be quick, safe, and effective in guiding injections [243]; however, it may not be necessary for parotid injections and may add value for submandibular injections [223]. In our practice, we commonly use BoNT for management of drooling in PD and have good results.

### 2.5. Tics

Tics are recurrent, jerk-like, or transiently sustained involuntary movements (motor tics) or noises (phonic tics) that occur abruptly out of background of normal activity [256]. BoNT was first used for the treatment of tics in a series of 10 patients with Tourette’s syndrome with focal tics and was found to be effective not only in controlling the tics but also in markedly reducing or eliminating the premonitory urge [257]. On 7 September 2020, when “botulinum” and “tics” was searched as title words, 15 studies that were in English and were human studies were found. Of the 15 articles, 2 were review articles, 3 were either prospective or retrospective trials, 1 were RCTs, 5 were case reports, 2 were unavailable for review, and 2 were commentary. Table 9 lists the RCT associated with tics and BoNT.

In one of our series, 35 patients with motor tics associated with Tourette’s syndrome received BoNT, and 82% experienced relief of tics and of premonitory urge with an average duration of benefit lasting 14.4 weeks [258]. In a series of 15 patients with motor tics, BoNTA was useful in 89% of patients and was found to be particularly helpful in the treatment of eye blinking and head jerking due to contractions of cervical muscles [259].

In 30 patients with phonic tics related to Tourette’s syndrome who received BoNTA, 93% noted meaningful improvement of symptoms [261] lasting mean of 102 days. Before BoNT injection, 53% of patients had premonitory urges as compared to 20% after BoNT treatment; 80% had hypophonia [261]. We and others reported that BoNT improves not only simple phonic tics but also complex phonic tics manifested by coprolalia [262,263]. A comprehensive review of BoNT in tics in Tourette’s syndrome, identified in 1 RCT with 18 patients with motor tics, concluded that they were not sure about the effects of BoNT on tics [260].

### 2.6. Myoclonus

Myoclonus is an involuntary, jerk-like contraction of muscle. If there is active muscle contraction, it is referred to as “positive myoclonus”, but an abrupt brief lapse of muscle contraction, as occurs in asterixis, is a characteristic feature of “negative myoclonus” [264]. Based on anatomical distribution and various physiologic characteristic, myoclonus is categorized as focal, segmental, or generalized or cortical, reticular reflex, and spinal myoclonus, but there are other classifications based on electrophysiological studies [265]. Though there are no randomized controlled trials, there are some case reports and series discussing use of BoNT for as a treatment of myoclonus [264,266,267]. On 7/12/2020, “myoclonus” and “botulinum” were searched for as title words. The results showed 21 articles; of these, 17 were in English and human studies. Of the 17 articles, 2 were either prospective or retrospective trials, 0 were RCTs, 13 were case reports, and 2 were commentaries.

A case series of nine patients with focal, segmental, and generalized myoclonus (due to variety of primary pathologies including trauma, astrocytoma, Lafora’s disease, and mitochondrial dysfunction) were reported to be responsive to BoNT injections, with only one patient reporting subjective arm weakness [264]. Case reports have suggested success in treating myoclonus associated with Rasmussen encephalitis [266], stimulus-sensitive myoclonus [268], and, in 3 patients, limb myoclonus after peripheral nerve injury [269].

Palatal myoclonus, also called palatal tremor, consists of involuntary contractions of the soft palate, producing rhythmic movement at a frequency of 1–10Hz, often accompanied by a clicking sound [270,271]. It may be accompanied by rhythmical movements of branchial and other muscles resulting in slow tremor, referred to as myorhythmia [272]. Palatal myoclonus has been usually attributed to a dysfunction or lesion in the Guillain–Mollaret triangle, but there are many other causes, including functional (psychogenic) causes, of this form of segmental myoclonus.

There are many case reports and series of BoNT being effective in the treatment of palatal myoclonus [267]. A retrospective series between 1985 to 2011 included 15 patients with essential palatal myoclonus in which 2.5 or 5 units of onabotulinumtoxinA produced amelioration of symptoms in 85.7% of cases [271].

### 2.7. Restless Legs Syndrome

Restless legs syndrome is a chronic or intermittent neurologic condition characterized by the urge to move lower limbs due to an uncomfortable sensation in the legs; the estimated prevalence in the general population is 5–15% [273]. RLS is more common in women and is often associated with positive family history, although no causative gene or genes have been identified. The pathophysiology of RLS is not yet fully understood, but disinhibition of motor pathways and dysfunctional sensory input to the cortex has been proposed [274]. Although dopaminergic agonists, α-2-δ calcium channel agents, such as gabapentin, and opioids are helpful, about 45% of patients continue to have bothersome symptoms after treatment [275]. BoNT injections have been reported to be helpful in some patients with RLS, particularly those who have disabling symptoms despite optimal medical treatment [6,14].

There have been several open-label trials [276,277] and case series [278] that have studied response of RLS to BoNT. A case series with three patients [277] and another case series with 27 patients [276] failed to show improvement of RLS symptoms with BoNT. An observational case series with three patients had concluded that intramuscular BoNTA injection improved RLS symptoms [278]. Mittal et al. [275] found a positive effect of BoNT on RLS symptoms, but Nahab et al. [279] found a lack of benefit in their series. Though there is conflicting evidence for efficacy of BoNT in RLS, we used it in a selected group of patients with “malignant” RLS. While not necessarily completely effective in all patients, most have benefited for about 3–4 months after injection, with BoNT targeting muscles that the patient identifies as particularly troublesome because of intense feeling of restlessness. The treatment must be individualized, but we typically inject about 100–300 U of onabotulinumtoxinA in the quadriceps or hamstrings and/or 50–100 units into gastrocnemius or posterior tibialis.

We conducted a PubMed search on 21 April 2020; using botulinum and restless as title words, a total of six articles were identified. All six of these were in English and were human studies. Of the six articles, three were either prospective or retrospective trials, two were RCTs, and one was a review article. Table 10 lists RCTs associated with RLS and BoNT.

### 2.8. Central Effects of BoNT

In addition to Ach, BoNT also blocks the release of other neurotransmitters, including adenosine triphosphate, substance P, and calcitonin-gene-related peptide. BoNT also downregulates sensory receptors, such as transient receptor potential cation channel subfamily V member 1 (TRPV1), also known as the capsaicin receptor and the vanilloid receptor 1, and purinergic (P2X3) receptors [280]. BoNT’s effects on these transmitters and receptors is being explored in the management of autonomic and pain disorders such as those associated with cancer [281].

A variety of animal and human physiological and imaging studies have provided evidence that BoNT not only acts peripherally but also centrally; it is beyond the scope of this article to review all the evidence, but the reader is referred to some recent reviews on this topic [282,283,284]. Finally, there is emerging evidence that BoNT blocks the transsynaptic transmission of alpha-synuclein [285], the rogue protein involved in the pathogenesis of Parkinson’s disease and other neurodegenerative disorders [286,287].

## 3. Conclusions

BoNT is a safe and powerful treatment strategy for a variety of hyperkinetic movement disorders. The indications have gradually expanded over the last four decades, making BoNT one the most versatile drugs in the world. With advancing research into mechanisms of action, improved methods of administration, and novel formulations, the field of therapeutic BoNT will continue to grow [14,288].

## 4. Methods

A comprehensive review was conducted for select topics in movement disorders and use of BoNT injections in those conditions. Specific terms were used to search the PubMed database for articles in English, available in full, limited to human studies that were randomized controlled trials (identified based on PubMed filter). If the RCT was still ongoing or was not directly related to the use of BoNT in movement disorders, it was excluded. The title words used of the PubMed and the split-up of the articles identified is presented in a table form in each condition under the Results section. Relevant prospective and retrospective studies and RCTs in a table form were presented in the discussion.

## Figures and Tables

**Table 1:** Lists randomized controlled trials (RCTs) associated with blepharospasm.

Study	Study Design and Goal	Sample Size and Method	Results
Lungu et al., 2013 [56]	Double-blind placebo controlled randomized trial Formulation: onabotulinumtoxinA or rimabotulinumtoxinB B Assessed topical use of acetyl hexapeptide-8 (AH8), competitive SNAP25 inhibitor as new therapy for BSP	*n* = 24 Injections into OOc palpebral portion and 3/4 also received in the orbital portion/procerus/corrugator Topical application of AH8 or placebo started on Day 1 of BoNT	Time for symptom return to baseline was 3.7 and 3 months in active and placebo groups, respectively AH8 is safe and may be useful in increasing duration of effect
Wabbels et al., 2011 [57]	Double-blind, randomized trial Goal: compares incobotulinumtoxinA (Merz, BoNTA) to onabotulinumtoxinA	65 BSP patients received either incobotulinumtoxinA or onabotulinumtoxinA 20–40 U/eye Mean dose was 29 U/eye and 27 U/eye for onabotulinumtoxinA and incobotulinumtoxinA	Though there was no significant difference between the two formulations, however, there was a greater tendency to improve at 4 and 8 weeks with onabotulinumtoxinA BDSI score mean change was −0.42 in onabotulinumtoxinA and −0.21 in incobotulinumtoxinA No significant differences in side effects
Boyle et al., 2009 [58]	Prospective, randomized trial Study looks at differences between low (10 U/mL) and high (100 U/mL) concentrations of BoNTA	16 patients 10–30 units per side. Left and right sides were randomized	62% had equal relief of both sides No difference in efficacy Bruising and redness similar between groups
Truong et al., 2008 [59]	Randomized trial, double-blind, placebo controlled Studied safety and efficacy of abobotulinumtoxinA versus placebo in BSP	10, 23, 25, and 27 patients completed placebo, abobotulinumtoxinA 40, 80, and 120 U/eye, respectively	25%, 87%, 97%, and 94% of patients found placebo, 40 U/eye, 80 U/eye, and 120 U/eye to be effective Ptosis occurred in 4%, 13%, 39%, and 58% in placebo, 40 U/eye, 80 U/eye, and 120 U/eye, respectively Blurred vision occurred in 4%, 23%, 19%, and 42% in placebo, 40 U/eye, 80 U/eye, and 120 U/eye, respectively 80 U/eye provided the most efficacy while balancing for adverse effects.
Roggenkamper et al., 2006 [60]	Double-blind, randomized trial Assessed NT201(Merz Pharmaceuticals GmbH, Germany) new formulation of BoNTA compared to onabotulinumtoxinA	148 patients received NT201 and 152 patients received onabotulinumtoxinA Mean total dose in NT201 was 39.6 units and onabotulinumtoxinA 40.8 units	Mean change in Jankovic rating scale was −2.67 and −2.90 for onabotulinumtoxinA and NT201, respectively. NT201 is safe and efficacious for BSP treatment Ptosis, xerophthalmia, and abnormal vision occurred at 6.1%, 2%, and 1.4% in NT 201 Ptosis, xerophthalmia, and abnormal vision occurred in 4.5%, 0%, and 3.2% in the onabotulinumtoxinA group, respectively.
Mezaki et al., 1999 [61]	Double-blind trial Assesses effectiveness of type A versus type F versus combination of A+F	54 patients had 5 units each of A+F on one side and on the other side had A or F Patient were randomly given either type A or F on one side and the mixture in the other side	There was no difference between the groups when comparing the AF side The peak effect was similar among the three groups Duration of action of AF group was less than that of A and more than that of F alone.
Nussgens Z et al., 1997 [62]	Double-blind trial Studies onabotulinumtoxinA versus abobotulinumtoxinA	212 BSP patients received either onabotulinumtoxinA or abobotulinumtoxinA the first time and the other BoNT during second session OnabotulinumtoxinA average dose was 45.4 IU and abobotulinumtoxinA was 182 IU	OnabotulinumtoxinA and abobotulinumtoxinA lasted 7.98 ± 3.8 weeks and 8.03 ± 4.6 weeks, respectively Adverse effects such as ptosis, blurred vision, diplopia, hematoma, and tearing occurred in 17% and 24.1% of onabotulinumtoxinA and abobotulinumtoxinA, respectively (*p* < 0.01) Bioequivalence of onabotulinumtoxinA:abobotulinumtoxinA is 1:4 in this trial
Jankovic et al., 1988 [63]	Randomized double-blind placebo-controlled trial (after initial open-label phase) Assessed BoNTA for management of various focal dystonia including BSP	22 patients with focal dystonia received either BoNTA or saline	All 12 BSP patients had relief of symptoms with BoNT and none who received saline improved Mean beneficial effect lasted 12.5 weeks
Mitsikostas et al., 2020 [64]	Randomized double-blind placebo-controlled trial (following which there was an open-label phase) Assessed incobotulinumtoxinA for management of BSP	61 BSP patients were randomized to receive either 50 U or 25 U of incobotulinumtoxinA or placebo	A statistically significant improvement was noted in Jankovic rating scale in the 50U group compared to the placebo group Low adverse event rate of 22.2–42.1 was noted.
Sane et al., 2019 [65]	Triple masked randomized controlled trial Assessed efficacy of onabotulinumtoxinA versus Neuronox in BSP	24 patients with BSP were randomized to receive either formulation	Mean duration of improvement was 3.78 months Neuronox and onabotulinumtoxinA were similar in safety and efficacy

Abbreviations: BSP—Blepharospasm; BoNT—Botulinum toxin; BoNTA—Botulinum toxin A; OOc—Orbicularis oculi.

**Table 2:** Lists RCT associated with bruxism and BoNT.

Study	Study Design and Goal	Method	Results
Ondo et al., 2018 [82]	Randomized double-blind placebo-controlled trial Assessed onabotulinum toxinA for bruxism	*n* = 31 They were given either 200 U of BoNTA (60 and 40 in each masseter and temporalis) or given placebo	Total sleep time and bruxism episodes seemed to favor BoNTA Other than two patients noticing a change in how they smile, no significant side effects were noted
Jadhao et al., 2017 [83]	Randomized placebo-controlled trial Assessed BoNTA for treatment of pain in bruxism	*n* = 24 Patients were given either bilateral BoNTA or saline or no injections. Each group had eight patients	Pain improved in BoNTA, however, did not change in the other two groups
Shim et al., 2014 [84]	Randomized prospective trial Assessed BoNTA for motor contractions in sleep bruxism	*n* = 20 One group got 25 U in each masseter (*n* = 10) while the other group got injection in masseter and temporalis (*n* = 10)	The masticatory muscle activity frequency was unchanged, but the amplitude was lower Four weeks after injection, nine patients felt reduced teeth grinding and 18 felt reduced morning jaw stiffness
Lee et al., 2010 [85]	Double-blind randomized placebo-controlled trial	*n* = 12 Six patients received BoNT into each masseter while the other six received saline	Bruxism was lower in patients who received BoNT (*p* = 0.027)

Abbreviations: BoNT—Botulinum toxin; BoNTA—Botulinum toxin A.

**Table 3:** RCTs associated with cervical dystonia (CD) and botulinum.

Study	Study Design and Goal	Method	Results
Hu et al., 2019 [131]	Randomized trial Assesses effects of physical therapy (PT) on CD	16 CD and 10 healthy 16 CD were randomly assigned to pure BoNT or BoNT with PT (this group received BoNT and PT for 6 weeks)	Toronto Western Spasmodic Torticollis Rating Scale (TWSTRS) score severity and pain improved by 31% and 28% in BoNT-PT arm PT can be used as adjunct for CD
Yi et al., 2018 [132]	Randomized, double-blind, placebo-controlled Assesses efficacy and safety of BoNTA in CD in dyskinetic cerebral palsy	16 patients with dyskinetic cerebral palsy were injected with either BoNTA or saline	At 4 weeks, TWSTRS total score improved in BoNTA as compared to saline (*p* = 0.028). Dysphagia occurred in two patients in BoNTA arm and one in saline arm BoNTA for CD in dyskinetic CP is safe and helps with pain and disability
Samotus et al., 2018 [133]	Randomized prospective trial Assesses if kinematic-based (KB) muscle selection for BoNT has better outcomes than visual-based selection (VB) for BoNT in treatment of CD	28 CD patients were divided in either VB or KB group Injections were performed at 0, 16, and 32 weeks with follow-up after 6 weeks of each injection	TWSTRS score in VB score significantly reduced by 28.5% only after second injection, but the score in KB group reduced by 28.8% at week 6 KB can lead to quicker muscle selection
Huang et al., 2015 [134]	Randomized prospective trial Studies efficacy of ultrasound-guided injection of BoNTA for CD BoNTA by Lanzhou Institute of Biological Products was used.	105 patients were divided in three groups They either received oral medications (trihexyphenidyl, diazepam, haloperidol baclofen, carbamazepine) or BoNTA under US guidance or BoNTA under US guidance with orthopedic brace	No differences were noted in Tsui and Spitzer score in medication group The Tsui and Spitzer scores in the BoNTA and BoNTA with orthopedic brace groups were improved Tsui score in BoNTA with brace was 5.8 ± 3.7 at 3 months as compared to 8.6 ± 3.4 for BoNTA Using orthopedic brace with BoNTA can lower muscle spasm and QoL
Comella et al., 2011 [135]	Prospective, double-blind, randomized placebo-controlled trial Compares incobotulinumtoxinA to placebo in CD	233 patients with CD were randomly assigned 1:1:1 to placebo or incobotulinumtoxinA 120 U or incobotulinumtoxinA 240 U	TWSTRS total score change from baseline was -2.2, –9.9, and –10.9 for placebo, incobotulinumtoxinA 120 U, and incobotulinumtoxinA 240 U, respectively Dysphagia, neck pain, and muscle weakness occurred at 2.7%, 4.1%, and 1.4% of control; 11.5%, 5.1%, and 6.4% of incobotulinumtoxinA 120 U, and 18.5%, 14.8%, and 11.1% of incobotulinumtoxinA 240 U IncobotulinumtoxinA is safe and useful
Truong et al., 2010 [136]	Randomized, double-blind, placebo-controlled Assessed safety and efficacy of abobotulinumtoxinA for managing CD	55 CD patients received abobotulinumtoxinA and 61 placebo Starting dose of abobotulinumtoxinA was 500 units 55 CD patients received abobotulinumtoxinA and 61 placebo	TWSTRS total score decreased to −15.6 ± 2 and −6.7 ± 2 in abobotulinumtoxinA and placebo groups AbobotulinumtoxinA has good safety and efficacy
Quagliato et al., 2010 [137]	Prospective, randomized, double-blind trial Compares Prosigne and onabotulinumtoxinA	24 patients were randomly assigned to get either 300 U of onabotulinumtoxinA or Prosigne Depending on cervical dystonia, muscles were selected	OnabotulinumtoxinA and Prosigne have 1:1 safety and adverse effect profiles
Pappert EJ et al., 2008 [138]	Randomized, double-blind trial Compares BoNTA versus BoNTB for CD	*n* = 111 55 CD patients received BoNTA and 56 received BoNTB	Total TWSTRS score decreased by 11 and 8.8 for BoNTB and BoNTA, respectively Injection site pain and trouble swallowing was similar in two groups Dry mouth was more in BoNTB (39.3%) while it was 7.3% in BoNTA Both formulations are effective for CD patients
Tassorelli et al., 2006 [139]	Randomized crossover trial Assesses BoNTA versus BoNTA with physical therapy for CD	*n* = 40 40 CD patients were randomly given either BoNTA or BoNT-PT, then had crossover Max dose was 500 U/pt	Duration of improvement was 118.8 days in BoNT-PT and 99.1 days in BoNT arm Disability with activities of daily living and pain were improved by BoNT-PT PT with BoNT would be helpful for CD
Comella et al., 2005 [140]	Randomized double-blind trial Assesses BoNTA versus BoNTB in CD	*n* = 139 74 patients received BoNTA (maximum dose 250 U) and 65 BoNTB (max dose 10,000 U)	TWSTRS score improvement was similar between groups: 9.3 with BoNTA and 10.2 with BoNTB Dysphagia and dry mouth significantly lower was lower in BoNTA; duration of effect was longer in BoNTA (14 weeks)
Truong et al., 2005 [141]	Double-blind, randomized trial Assessed abobotulinumtoxinA safety and efficacy in CD in U.S.A.	*n* = 80 They were randomly given either 500 units abobotulinumtoxinA or placebo	38% of abobotulinumtoxinA and 16% of placebo had benefit Mean duration of effect of abobotulinumtoxinA is 18.5 weeks Blurred vision (14%) and weakness (11%) was more in abobotulinumtoxinA
Benecke et al., 2005 [142]	Randomized double-blind trial Compared NT201 to OnabotulinumtoxinA in treatment of CD	*n* = 463 70–300 U either formulation was injected in patients with CD	Both had median TWSTRS severity score of 18 and improved to −6.6 in NT201 and −6.4 in onabotulinumtoxinA group 28.1% in NT201 and 24.1% in onabotulinumtoxinA group had adverse effects Safety and tolerability were alike in both groups
Laubis-Herrman et al., 2002 [143]	Randomized double-blind trial Efficiency of low-dose BoNT was studied in CD	31 patients with CD with at least two prior abobotulinumtoxinA injections were given either 547 ± 113 or 130 ± 32 mouse units of abobotulinumtoxinA	At 4 weeks, both groups showed similar decrease in TWSTRS score Duration of effect was 65.8 and 57.4 days in high- and low-dose groups
Naumann et al., 2002 [144]	Randomized double-blind trial, crossover design Compared original onabotulinumtoxinA versus current onabotulinumtoxinA for CD treatment	*n* = 133 100–300 U were injected per session Mean dose injected was 155 U and 156 U for original and current, respectively (Splenius capitis, SCM, trapezius, levator scapulae, scalene, platysma, zygomaticus, semispinalis, and paravertebral)	TWSTRS score improved by −5.34 and −6.20 with original and current onabotulinumtoxinA 53% and 52% of original and current onabotulinumtoxinA had adverse effects Adverse effects were dysphagia, neck pain, headache, asthenia, and pain at onabotulinumtoxinA site and weakness of muscle. The efficacy and safety of original and current onabotulinumtoxinA is similar
Wissel et al., 2001 [145]	Prospective, double-blind placebo-controlled parallel trial Assesses safety and efficacy of abobotulinumtoxinA 500 units in CD patients with Tsui score ≥9	*n* = 68 68 patients were randomized to receive either placebo or abobotulinumtoxinA	49% of abobotulinumtoxinA and 33% of placebo patients had improvement in pain. 86% and 42% of abobotulinumtoxinA and placebo patients were categorized as responders 42.9% and 27.3% of abobotulinumtoxinA and placebo had adverse effects. Neck weakness occurred only in abobotulinumtoxinA group 500 units of abobotulinumtoxinA is safe and effective for CD
Whitaker J et al., 2001 [146]	Randomized prospective trial BoNTA was used Assesses safety and efficacy of BoNT injected by outreach nurse practitioner as compared to outpatient hospital administered BoNT for BSP, CD, and HFS	*n* = 89 45 patients received home treatment while 44 received clinic treatments	Adverse effects between two groups were similar (except dysphagia was greater in the clinic group) Trained nurse injections were similar in efficacy and adverse events
Brashear A. et al., 1999 [147]	Randomized double-blind placebo-controlled trial Assessed BoNTB for CD (who were responding to BoNTA)	*n* = 109 109 patients were given either placebo or 5000 U BoNTB or 10,000 U BoNTB	Adverse effects were comparable between the three groups TWSTRS severity score did not significantly improve in the 5000 U group but was significant in 10,000 U group (*p* = 0.0016) NeuroBloc (BoNTB) is safe and useful at 5000 U and 10,000 U
Brin et al., 1999 [148]	Randomized double-blind placebo-controlled trial Assessed BoNTB in CD patient who were resistant to BoNTA	*n* = 77 38 patients received placebo and 39 received BoNTB	TWSTRS total score improved at Weeks 4, 8, and 12 Dry mouth was more common in the BoNTB arm In BoNTA-resistant CD, BoNTB (NeuroBloc) was safe and effective.
Brans et al., 1998 [149]	Assesses EMG features in CD after BoNTA Double-blind randomized trial	*n* = 42 EMG performed in 42 CD patients pre- and 4 weeks post- either BONTA or trihexyphenidyl	They concluded that EMG-guided application of BoNTA might be useful
Poewe et al., 1998 [150]	Randomized placebo-controlled double-blind dose ranging study in CD	*n* = 75 75 CD patients either received placebo or Dysport (250 vs. 500 vs. 1000 units) in ipsilateral splenius capitis and contralateral sternocleidomastoid	Adverse effects were greater in 1000 U compared to placebo and 250 U Good efficacy with low side effects were observed in 72%, 44%, 39%, and 10% of 1000 U, 500 U, 250 U, and placebo, respectively
Lew et al., 1997 [151]	Assessed BoNTB in BoNTA responsive and resistant CD patients	*n* = 122 They received either placebo or 2500 U or 5000 U or 10,000 U of BoNTB	TWSTRS total score was more in all three dosages of BoNTB and response increased with higher doses Dry mouth and dysphagia were the most common adverse effects BoNTB is safe and useful for CD management.
Brans et al., 1996 [152]	Randomized double-blind prospective trial Assesses effectiveness of BoNTA versus trihexyphenidyl for CD treatment	*n* = 64 32 received BoNTA mean dose 292 (1st injection) with placebo tablets, and the other 32 got trihexyphenidyl with placebo injection	BoNTA was more effective in treatment of CD
Ostergaard et al., 1994 [153]	Turns–amplitude analysis on EMG-guided BoNT for CD	*n* = 19 10 patients received BoNT and 9 received placebo	BoNT was effective at 89%; quantitative EMG is helpful for picking overactive muscles for BoNT
Yun et al., 2015 [154]	Randomized double-blind crossover trial Assessed abobotulinumtoxinA versus onabotulinumtoxinA at 2.5:1 ratio in management of CD	103 patients were randomly given one of the formulation for 16 weeks, and then, after 4 week washout, were given the other formulation for another 16 weeks	AbobotulinumtoxinA given at ratio of 2.5:1 compared to onabotulinumtoxinA had similar efficacy and adverse effect profile

Abbreviations: BoNT—Botulinum toxin; BoNTA—Botulinum toxin A; BoNTB—Botulinum toxin B; CD—Cervical dystonia; EMG—Electromyography; PT—Physical therapy; TWSTRS—Toronto Western Spasmodic Torticollis Rating Scale.

**Table 4:** RCTs associated with limb dystonia and botulinum.

Study	Study Design and Goal	Sample Size and Method	Results
Umar M et al., 2018 [172]	Randomized trial Assessed the effect of BoNTA and task-specific training in post-stroke focal dystonia	23 patients in experimental group got BoNTA and task-specific training and 23 in control group got only task-specific training Different muscles in upper limb were injected based on type of dystonia	Motor assessment scale improved in both; however, no significance variations were noted between the two groups Task-specific training is helpful for post-stroke dystonia
Geenen et al., 1996 [173]	Randomized prospective trial Assessed efficacy of BoNT for focal dystonia identified through either EMG with or without stimulation for focal hand dystonia	*n* = 12 Eight and four patients received BoNT in target muscle based on EMG guidance without and with stimulation	4 and 3 in the without and with stimulation group had weakness in target muscle EMG with stimulation is at least as effective as EMG without stimulation

Abbreviations: BoNT—Botulinum toxin; BoNTA—Botulinum toxin A; EMG—Electromyography.

**Table 5:** Lists the RCTs identified in hemifacial spasm (HFS).

Study	Study Design and Goal	Method	Results
Xiao et al., 2018 [198]	RCT Formulation used: Chinese BoNTA (CBTXA—Lanzhou Biological Products Institute, Lanzhou, China) or onabotulinumtoxinA Evaluates if facial asymmetry improved with bilateral BoNT	19 HFS patients received unilateral BoNT (UBT) 24 HFS patients received bilateral BoNT Injected in orbicularis oculi (OOc), zygomaticus major (ZM), risorius (ris), orbicularis oris (OOr), levator labii superioris (LLS), frontalis, and glabella, respectively In patients who got bilateral injections, affected side was injected the same as UBT (1.25–4 MU) and unaffected side same muscles were injected with 1.25MU.	Bilateral facial injections reduced facial asymmetry Unilateral BoNT injections increased asymmetry BoNT duration of effect and adverse effect were similar between both groups
Lolekha et al., 2017 [34]	Double-blinded, cross RCT Formulation used: onabotulinumtoxinA Compares preseptal versus pretarsal BoNT injections in treatment of HFS and BSP	40 (31 HFS, 9 BSP) 20 patients were in each arm and then had crossover If BSP, medial and lateral segments of upper and lower eyelid with up to 10 units per eye lid. If HFS, additional dose was injected in OOc, ZM, LLS, and mentalis. Total dose was 12.5–22.5 units and 5–10 units per eye lid	Pretarsal BoNT had improved symptom control, decreased latency to improvement, and increased duration of efficacy. Adverse effects of ptosis were seen in the preseptal group only However, minor complications such as hematoma, tearing, and irritation was seen in both groups
Li et al., 2015 [199]	Randomized, double-blind, crossover trial Formulation: BoNTA Assesses differences in low (25U/mL) versus high (50 U/mL) BoNT for treatment of HFS	*n* = 20 2.5 to 5 U were injected in each location	Time of onset of efficacy was not significantly different The high concentration group had longer duration of efficacy 15/20 and 4/20 of high and low concentration groups, respectively, had adverse effects. The adverse effects lasted longer in the high concentration group
Prutthipongsit et al., 2015 [200]	Randomized, double-blind trial Formulation used: abobotulinumtoxinA Efficacy differences between split and nonsplit site injection of BoNT for HFS	31 HFS (16 patients in split site and 15 in nonsplit site) Nonsplit site—ZM and ris were injected in two spots in each (total four spots). Onespot in each muscle got a full dose of abobotulinumtoxinA and the other spot in each muscle got saline Split site—ZM and zygomaticus minor (Zmi) and two injections in risorius (ris). All four spots received abobotulinumtoxinA	Median onset of efficacy was 4 and 4.5 days for nonsplit and split, respectively Duration of effect was 60 days and 54.5 days for nonsplit and split, respectively The efficacy was similar between both groups
Li et al., 2012 [55]	Randomized controlled trial Formulation used: obtained from Lanzhou Institute of Biological Products Efficacy of BoNTA versus BoNTA with Carbamazepine (CBZ) was assessed	58 patients with either HFS or BSP were randomized 30 patients got BoNTA with CBZ 100 mg 3 times a day and 28 received only BoNTA 4–5 injections per eye, 2.5 to 5 U per site, and addition 3 sites in face for HFS. Up to 55 U for BSP and 75 U for HFS	Complete remission was noted in 90% and 67.9% of treatment and control groups The duration of effect was similar between the 2 groups
Kongsengdao et al., 2012 [201]	Prospective, double-blinded, randomized, crossover trial Evaluates quality of life (QoL) of Neuronox and abobotulinumtoxinA	26 HFS patients were randomly divided into Neuronox or abobotulinumtoxinA group and switched at 12 weeks Four injections around OOc and one each in upper and lower OOr with either 15 units of abobotulinumtoxinA or 3.125 units of Neuronox	The mean QoL scales (HFS-30, SF-36, AIMS) was not significantly different between 2 groups The total intensity score of HFS was significantly lower in the abobotulinumtoxinA group QoL scores were similar between abobotulinumtoxinA and Neuronox
Wu et al., 2011 [52]	Prospective, open-label and randomized Compares efficacy of CBTXA and onabotulinumtoxinA in HFS and BSP	273 patients with HFS and BSP 107 received onabotulinumtoxinA and 166 CBTXA Per injection site dose was 2.5–5 U	Both formulations have similar efficacy Response rate 97% with CBTXA and 99% with onabotulinumtoxinA Most common adverse effect was tightness in face and facial droop
Colakoglu et al., 2011 [202]	Randomized, single-blind, cross over trial Formulation: onabotulinumtoxinA Assessed efficacy of upper and lower facial versus pure upper facial BoNTA injections for HFS	23 patients with HFS were randomized to receive BoNTA in both OOc and perioral muscles versus BoNTA in OOc and physiological serum into perioral muscles 11–30 units of onabotulinumtoxinA in OOc and 3–11 units in lower facial (four points in ZM, Zmi, LLS, and ris)	When patients had more severe HFS symptoms in lower face, receiving BoNTA in perioral muscles was more effective Adverse effects of moderate/mild lower facial paresis were 13%/34.7% and 0%/13% in BoNT only group versus BoNT group
Quagliato et al., 2010 [53]	Prospective, randomized, double-blind trial Compares efficacy of Prosigne (Chinese origin BoNTA) and onabotulinumtoxinA	36 HFS and 21 BSP were randomized to receive either of the formulations BSP—30 units were injected in each OOc and procerus HFS—25 U OOc, 10 U spread among in ZM, Zmi, LLS, ris, levator anguli oris, and depressor anguli, mentalis, and platysma	Duration of effects was 11.3 weeks with both forms in BSP and 12.8/12.9 weeks onabotulinumtoxinA/Prosigne for HFS OnabotulinumtoxinA and Prosigne have similar efficacy and adverse effects
Price et al., 1997 [54]	Randomized, prospective trial Determines effectiveness of four sites of injection of BoNT with most efficacy and least side effects (for BSP and HFS)	92 (50 BSP and 42 HFS) Four different protocol of injections were studied with four spots of injections in OOc in each (standard, brow, and inner orbital or outer orbital) (HFS had addition cheek injection on affected side) 2.5 units were injected in four sites around eye	Standard treatment had longest effect in BSP, and brow treatment was preferred treatment for HFS Inner orbital had more ptosis in BSP group Outer orbital had lowest duration of benefit
Yoshimura et al., 1992 [203]	Prospective randomized placebo-controlled Formulation: Oculinum (Alan Scott, MD, Smith Kettlewell Eye research institute) Assessed efficacy of BoNT for HFS	11 HFS patients Three different doses (2.5–10 units) of Oculinum or normal saline (placebo) was injected in a random manner	84% of BoNT provided relief and 44% was substantial Side effects were facial weakness (97%), bruising (20%), double vision (13%), and drooping eye lid (7%) BoNT was safe and effective for treatment of HFS

Abbreviations: BSP—Blepharospasm; BoNT—Botulinum toxin; BoNTA—Botulinum toxin A; BoNTB—Botulinum toxin B; CBZ—Carbamazepine; CD—Cervical dystonia; CBTXA—Chinese Botulinum toxin A; EMG—Electromyography; HFS—Hemifacial spasm; LLS—Levator labii superioris; RCT—Randomized controlled trial; Ris—Risorius; UBT—Unilateral botulinum toxin; Zmi—Zygomaticus minor; ZM—Zygomaticus major.

**Table 6:** RCTs in tremors and BoNT.

Study	Study Design and Goal	Method	Results
Mittal et al., 2018 [216]	Randomized, double-blind placebo-controlled, prospective crossover trial Assessed safety and usefulness of incobotulinumtoxinA for management of essential tremor (ET)	*n* = 33 Either placebo (normal saline) or 80–120 units of incobotulinumtoxinA with EMG guidance was injected in hand and forearm of patients with moderate to severe ET	Fahn Tolosa Marin score median comparison between incobotulinumtoxinA/placebo was 2 and placebo/incobotulinumtoxinA was 3 at week 8 Two patients in incobotulinumtoxinA group at hand weakness IncobotulinumtoxinA was found useful in improving tremor scores in patients with ET
Mittal et al., 2017 [217]	Randomized, double-blind placebo-controlled, prospective crossover trial Assessed safety and usefulness of incobotulinumtoxinA for management of PD tremor	*n* = 30 Patients either received placebo or 7–12 injections of incobotulinumtoxinA (total dose 85–110units, using EMG guidance). The lumbricals (97%), FCR (90%), FDS (87%), FCU, pronator, and biceps (83%) were the most commonly injected muscles.	UPDRS rest tremor (*p* < 0.001) and NIHCGC improved (*p* < 0.001) significantly at weeks 4 and 8. IncobotulinumtoxinA was found useful in improving PD tremor scores and patient symptoms.
Bertram et al., 2013 [218]	Randomized, double-blind placebo-controlled, prospective crossover trial Studied safety and efficacy of abobotulinumtoxinA for postural orthostatic tremor (POT)	*n* = 8 POT diagnosed with electrophysiology were randomized to receive either placebo or 200 units of abobotulinumtoxinA in tibialis anterior.	The tremor frequency remained unchanged. 200 units of abobotulinumtoxinA did not affect patient symptoms of unsteadiness and falls in POT.
Walt et al., 2012 [219]	Randomized double-blind crossover study	*n* = 23 Each limb was randomly assigned to either 100 units BoNTA (under EMG guidance) or placebo (0.9%) and the other treatment at 12 weeks.	Bain score improved after BoNT at 6 (*p* = 0.0005) and 12 weeks (*p* = 0.0001). Hand weakness was more common in BoNT group (42.2%) compared to placebo (6.1%). BoNTA can improve arm tremor in MS.
Adler et al., 2004 [220]	Randomized prospective study Assessed BoNTA for voice tremor management	*n* = 13 13 patients with voice tremor were randomized to receive either 1.25 or 2.5 or 3.75 U of BoNTA.	Mean time of onset of efficacy was 2.3 days; mean tremor severity score improved by 1.4 points at week 2. Dysphagia was a noted adverse event.
Brin et al., 2001 [213]	Randomized double-blind trial OnabotulinumtoxinA Evaluates BoNTA for ET of hand	*n* = 133 133 ET patients received 50U (*n* = 43) or 100 U (*n* = 45) or placebo (*n* = 45) under EMG guidance into FCR, FCU, ECR, and ECU. In 100 U = 30U FCR, 30U FCU, 20U ECR, 20U ECU In 50U = 15U FCR, 15U FCU, 10U ECR, 10U ECU	Postural component was lower after weeks 4 and 16, while kinetic component was lower at week 6. Grip strength was lower in high- and low-dose BoNT.
Jankovic et al., 1996 [212]	Randomized double-blind placebo-controlled trial Assesses BoNTA for essential hand tremor	*n* = 25 BoNTA or Placebo injected into wrist flexors and extensors	Tremors improved at 4 weeks (*p* < 0.05) compared to placebo. All BoNTA treated patients had finger weakness.
Pahwa et al., 1995 [221]	Randomized double-blind placebo-controlled trial Assess BoNT for essential head tremor	*n* = 10 10 patients with head tremor got either normal saline or BoNT under EMG guidance and had the other treatment after 3 months 40 U in each SCM and 60 U in each splenius capitis	Examiner 50% and 10% improvement in BoNT and placebo group They inferred that BoNT may be helpful if patients did not respond to oral medications.
Rajan et al., 2020 [222]	Randomized placebo-controlled trial Assessed BoNT in upper extremity dystonic hand tremor	*n* = 30 15 received placebo and 15 received onabotulinumtoxin A	Fahn–Tolosa–Marin tremor rating scale total score was lower in BoNT group at weeks 6 (*p* < 0.001) and 12 (*p* = 0.03).

Abbreviations: BSP—Blepharospasm; BoNT—Botulinum toxin; BoNTA—Botulinum toxin A; BoNTB—Botulinum toxin B; CD—Cervical dystonia; EMG—Electromyography; ET—Essential tremor; ECR—Extensor carpi radialis; ECU—Extensor carpi ulnaris; FCR—Flexor carpi radialis; FCU—Flexor carpi ulnaris; PD—Parkinson’s disease; POT—Postural orthostatic tremor; SCM—Sternocleidomastoid; UPDRS—Unified Parkinson’s disease rating scale; NIHCGC—National Institutes of Health Collaborative Genetic Criteria.

**Table 7:** Parkinson’s disease (PD)-related conditions amenable to treatment with BoNT.

Dystonia - BSP/lid apraxia - Bruxism - Limb dystonia - Cervical dystonia - Camptocormia - Levodopa-induced dyskinesia [225], discussed in table	Numerous studies have tried treating various dystonic symptoms in patients with Parkinson’s disease [224,226,227].
Jaw tremors	In three patients with PD jaw tremor who underwent Dysport injection, mean dose of 53 units into each masseter and improvement was noted in jaw tremor in all three patients without side effects [228].
Freezing of gait	Freezing of gait (FOG) is thought to be due to activation of both agonist and antagonist muscle in the legs, which is similar to pathophysiology of dystonia, hence studies have looked into botulinum for freezing of gait [229,230].
Sialorrhea	Increased drooling is seen in about 10% of PD [231] and multiple studies have looked at used of botulinum injection for sialorrhea [231,232].
Overactive bladder	In four PD and two MSA patients with overactive bladder (OAB) complaints, 200 U BoNTA was injected into detrusor, and all patients experienced relief of symptoms without systemic adverse effects [233]. Similar results were seen in eight PD patients with OAB post-BoNTA [234].
Constipation	In a study with PD patients with constipation (after excluding those related to slow movement in colon), in an open-label study, Botox was injected into puborectalis muscles and noted improvement in symptoms in 10 patients at 2 months [235].

BoNT—Botulinum toxin; BoNTA—Botulinum toxin A; MSA—Multiple system atrophy; PD—Parkinson’s disease; OAB—Overactive bladder.

**Table 8:** RCT associated with PD and BoNT.

Study	Study Design and Goal	Method	Results
Rieu et al., 2018 [244]	Double-blind randomized trial Assessed incobotulinumtoxinA for foot dystonia related to Parkinson’s disease	45 PD patients were injected with either 100UI incobotulinumtoxinA or placebo in flexor digitorum longus and brevis	Mean clinical global impression was better in the treatment group as compared to the placebo Pain and dystonia severity were reduced in the treatment group
Bruno et al., 2018 [245]	Randomized placebo-controlled double-blind crossover prospective trial Assessed BoNT for limb pain in PD	*n* = 12 BoNTA under EMG was used at average dose of 241.6 U	Temporary muscle weakness was seen in two patients (one in each group) BoNTA led to NRS score to drop significantly at week 4 (−1.75 points lower), whereas there was not a significant change in the placebo group
Narayanaswami et al., 2016 [232]	Randomized placebo-controlled double-blind crossover prospective trial Assessed incobotulinumtoxinA for treatment of drooling in PD	*n* = 9 Subjects were randomized to receive either 100 U of incobotulinumtoxinA or saline was injected into each submandibular (30 U) and parotid glands (20 U).	Saliva weight was similar between both groups pre- and postinjections One patient had difficulty chewing and swallowing while another had thicker saliva during the incobotulinumtoxinA injections In this study, incobotulinumtoxinA was not helpful for drooling in PD
Bonanni L et al., 2007 [246]	Randomized blinded crossover trial AbobotulinumtoxinA was used Assesses BoNT for lateral axial dystonia due to Parkinson’s disease	*n* = 9 with lateral axial dystonia due to Parkinson’s disease Four patients received BoNT and five got placebo, and five got placebo and then switched over after 3 months 500 units were injected in four paraspinal muscle sites	Six patients found BoNT to be effective, two had no change, and one had subjective improvement without change in lateral bending
Tassorelli et al., 2014 [247]	Randomized placebo-controlled double-blind prospective trial Assessed if BoNTA helped increase rehabilitation effects in PD patients with Pisa syndrome	*n* = 26 They were randomized to receive rehabilitation therapy with or without BoNTA (total dose 50–200 UI)	Patients who received rehabilitation therapy had better posture, but those who also received BoNTA had more pain reduction and longer improvement in clinical variables
Chinnapongse et al., 2012 [248]	Randomized placebo-controlled double-blind with sequential dose escalation Assessed BoNTB for sialorrhea in PD	*n* = 54 They were randomized and given either placebo or 1500U/2500U/3500U of BoNTB into submandibular (250 units for each side) and parotid glands	Dry mouth was seen in 15% of BoNTB patients. Drooling frequency and severity scale was better in BoNTB arm than placebo at four weeks (*p* < 0.05), and this was dose-dependent BoNTB is safe and effective for treatment of sialorrhea in PD
Espay et al., 2011 [225]	Double-blind crossover trial Assessed cervical BoNTA for treatment of levodopa-induced dyskinesia	*n* = 12 EMG-guided BoNTA or placebo was injected in neck muscles. SCM 25U, Splenius capitis 50U divided into each side, trapezius 25 U bilaterally	Four patients finished the 6-month trial There was a lack of positive effect. There was neck weakness
Guidubaldi et al., 2011 [249]	Randomized double-blind crossover trial Assessed BoNTA versus BoNTB for drooling in PD or ALS	*n* = 27 (15 ALS and 12PD) Either got BoNTA or BoNTB ultrasound-guided into parotid and submandibular glands Either 250 U of abobotulinumtoxinA (BoNTA) or Neurobloc 2500 U (BoNTB)	Latency to benefit was shorter for BoNTA (6.6 ± 4.1days) and BoNTB (3.2 ± 3.7days) Duration of effect was similar between both groups
Lagalla et al., 2009 [250]	Randomized double-blind placebo-controlled trial Assessed BoNTB for drooling in PD	*n* = 36 Patients either got 4000 U of BoNTB or placebo	Patients who received BoNTB noted 44.4% and 33.3% (moderate and dramatic) reduction in sialorrhea Useful effects lasted 19.2 ± 6.3 weeks in BoNTB-treated patients (*p* < 0.0001)
Kalf et al., 2007 [251]	Randomized prospective trial Compares BoNTA in submandibular versus parotid injections	*n* = 17 These patients either received 150 MU abobotulinumtoxinA divided between each gland, either submandibular or parotid	Two patients developed transient dysphagia (one in each group) Dry mouth was noted in three and one time after submandibular and parotid groups, respectively Within the submandibular group, DSFS and social consequences were improved. This was not seen in the parotid group 50% and 22% of patients in the submandibular and parotid groups were noted as responders
Lagalla et al., 2006 [252]	Double-blind randomized placebo-controlled study Assessed BoNTA for drooling in PD	*n* = 32 They received 50 U of onabotulinumtoxinA in each parotid or placebo	Patient that received BoNT had improved frequency of drooling and reduced social disability No adverse effects were reported
Wieler et al., 2005 [253]	Double-blind randomized placebo-controlled crossover study Assessed BoNTA for freezing of gait (FOG)	*n* = 12 Patients got either BoNTA or placebo and had crossover for five visits 200–300 U was given in the gastrocnemius and soleus under EMG guidance (up to 150 U per limb)	FOG did not improve after BoNT
Fernandez et al., 2004 [229]	Double-blind randomized placebo-controlled study Assessed BoNTB for FOG	*n* = 14 14 were randomly given either 5000 U of BoNTB (*n* = 9) or placebo (*n* = 5) Injections were in soleus and gastrocnemius	No difference noted in FOG between two groups
Dogu et al., 2004 [254]	Randomized prospective trial Assessed US-guided versus anatomically injected intraparotid BoNTA for drooling in PD	*n* = 15 Patients were randomly given either US-guided (*n* = 8) or blind (*n* = 7) onabotulinumtoxinA injections into parotid (15 U in each parotid)	Two patients in US-guided group had dry mouth Mean time to have lower saliva production was 4.1 days and duration of effect was about 4.4 months US guidance may be safe and easy to use
Ondo et al., 2004 [255]	Double-blind randomized placebo-controlled study Looks at BoNTB (rimabotulinumtoxinB) for drooling in PD	*n* = 16 They either received BoNTB (1000 U in each parotid or 250 U in each submandibular) or placebo.	Patients who got BoNT did improve on visual analogue scale (*p* < 0.001) and drooling scale (*p* < 0.05) BoNTB is effective for drooling in PD

Abbreviations: ALS—Amyotrophic lateral sclerosis; oNT—Botulinum toxin; BoNTA—Botulinum toxin A—BoNTB—Botulinum toxin B; DSFS—Drooling severity and frequency score; EMG—Electromyography; FOG—Freezing of gait; MSA—Multiple system atrophy; PD—Parkinson’s disease; Ultrasound—US.

**Table 9:** Lists the RCT associated with tics and BoNT.

Study	Study Design and Goal	Sample Size and Method	Results
Marras et al., 2001 [260]	Randomized double-blind placebo-controlled cross-over trial Formulation: onabotulinumtoxinA Assessed if BoNT was useful for simple motor tics	*n* = 18 Tics involving face, neck, or shoulder were selected for BoNT. Patients were either injected with onabotulinumtoxinA or placebo	Blinking and head turning were the most common tics treated Median proportional change in placebo was +5.8% and BoNT was –39% BoNT helped decrease the premonitory urge and the frequency of tics.

Abbreviation: BoNT—Botulinum toxin.

**Table 10:** Lists the two RCTs identified with RLS and BoNT.

Study	Study Design	Method	Results
Mittal, 2018 [275]	Double-blinded, placebo-controlled crossover randomized controlled trial (RCT) Formulation used: incobotulinumtoxinA	Sample size (*N*): 24 40 units each for tibialis anterior (TA), gastrocnemius (GCS) and 20 units in biceps femoris bilaterally Controls were injected with saline	International restless legs syndrome score improved at Week 4 (*p* = 0.0036) and Week 6 (*p* = 0.0325). No significant improvement at Week 8 (*p* = 0.067) They concluded that incobotulinumtoxinA injected improved RLS severity without adverse events
Nahab, 2008 [279]	Double-blinded, placebo-controlled crossover RCT Formulation used: onabotulinumtoxinA	*n* = 6 40 mU Quadriceps femoris (QF), 20 mU TA, 20 mU GCS, and 10 mU soleus (SOL) under EMG guidance Max dose: 90 mU/leg Controls were injected with saline	A statistically significant benefit was not noted, and adverse effects were similar with both groups

Abbreviations: EMG—Electromyography; GCS—Gastrocnemius; QF—Quadriceps femoris; RLS—Restless leg syndrome; RCT—Randomized controlled trial; SO—Soleus; TA—Tibialis anterior.

## Data Availability

The data cited in this article is based on previously published articles and relevant references have been quoted in the text.

## Abbreviations

AH8	Acetyl hexapeptide-8
AAN	American Academy of Neurology
ASD	Austim spectrum disorder
BSP	Blepharospasm
BSDI	Blepharospasm disability index
BoNT	Botulinum toxin
BoNTA	Botulinum toxin A
BoNTB	Botulinum toxin B
CBZ	Carbamazepine
CD	Cervical dystonia
CBTXA	Chinese botulinum toxin A
CGI	Clinical global improvement
CT	Computerized tomography
CBS	Corticobasal syndrome
DSFS	Drooling severity and frequency score
ET	Essential tremor
EMG	Electromyography
ECR	Extensor carpi radialis
ECU	Extensor carpi ulnaris
FF	Finger flexor
FCR	Flexor carpi radialis
FCU	Flexor carpi ulnaris
FOG	Freezing of gait
fMRI	Functional magnetic resonance imaging
GCS	Gastrocnemius
HFS	Hemifacial spasm
HD	Huntington’s disease
JRS	Jankovic Rating Scale
KB	Kinematic-based
LID	Levodopa-induced dyskinesia
LLS	Levator labii superioris
MHDA	Mouse hemidiaphragm assay
MPA	Mouse protection assay
MSA	Multiple system atrophy
NIHCGC	National Institutes of Health Collaborative Genetic Criteria
NAbs	Neutralizing antibodies
OOc	Orbicularis oculi
OOr	Orbicularis oris
OMD	Oromandibular dystonia
OMDQ-25	Oromandibular dystonia questionnaire
OAB	Overactive bladder
PD	Parkinson’s disease
POT	Postural orthostatic tremor
PT	Physical therapy
QoL	Quality of life
QF	Quadriceps femoris
RCT	Randomized controlled trial
RLS	Restless legs syndrome
Ris	Risorius
SNR	Secondary nonresponders
SOL	Soleus
SNARE	Soluble N-ethylmaleimide-sensitive factor attachment receptor
SNAP 25	25 kD synaptosomal-associated protein
SCM	Sternocleidomastoid
TA	Tibialis anterior
TWSTRS	Toronto Western Spasmodic Torticollis Rating Scale
US	Ultrasound
UPDRS	Unified Parkinson’s Disease Rating Scale
UBT	Unilateral botulinum toxin
FDA	United States Food and Drug Administration
VB	Visual-based
Zmi	Zygomaticus minor
ZM	Zygomaticus major
